# Malignant Course of Anomalous Left Coronary Artery Causing Sudden Cardiac Arrest: A Case Report and Review of the Literature

**DOI:** 10.1155/2015/806291

**Published:** 2015-07-15

**Authors:** Mahesh Anantha Narayanan, Christopher DeZorzi, Abhilash Akinapelli, Toufik Mahfood Haddad, Aiman Smer, Janani Baskaran, William P. Biddle

**Affiliations:** ^1^Department of Internal Medicine, CHI Health Creighton University Medical Center, 601 North 30th Street No. 5800, Omaha, NE 68131, USA; ^2^Creighton University School of Medicine, 2500 California Plaza, Omaha, NE 68102, USA; ^3^Cardiac Center of Creighton University, 3006Webster Street, Omaha, NE 68131, USA; ^4^Sri Venkateshwaraa Medical College Hospital and Research Center, Puducherry 605102, India

## Abstract

Sudden cardiac arrest has been reported to occur in patients with congenital anomalous coronary artery disease. About 80% of the anomalies are benign and incidental findings at the time of catheterization. We present a case of sudden cardiac arrest caused by anomalous left anterior descending artery. 61-year-old African American female was brought to the emergency department after sudden cardiac arrest. Initial EKG showed sinus rhythm with RBBB and LAFB with nonspecific ST-T wave changes. Coronary angiogram revealed no atherosclerotic disease. The left coronary artery was found to originate from the right coronary cusp. Cardiac CAT scan revealed similar findings with interarterial and intramural course. Patient received one-vessel arterial bypass graft to her anomalous coronary vessel along with a defibrillator for secondary prevention. Sudden cardiac arrest secondary to congenital anomalous coronary artery disease is characterized by insufficient coronary flow by the anomalous left coronary artery to meet elevated left ventricular (LV) myocardial demand. High risk defects include those involved with the proximal coronary artery or coursing of the anomalous artery between the aorta and pulmonary trunk. Per guidelines, our patient received one vessel bypass graft to her anomalous vessel. It is important for clinicians to recognize such presentations of anomalous coronary artery.

## 1. Introduction

Sudden cardiac arrest (SCA) is a known complication of congenital coronary anomalies. In a large registry of 126,595 patients undergoing coronary angiogram, the incidence of coronary anomalies was 1.3% [1]. About 80% of coronary artery anomalies are benign and incidental findings at the time of catheterization [1]. Younger patients in their first three decades with isolated coronary artery anomalies are at risk of dying, especially with exercise [2]. Potentially serious anomalies which include ectopic coronary origin from the pulmonary artery or opposite aortic sinus, single coronary artery, and large coronary fistulae can result in angina pectoris, myocardial infarction, heart failure, arrhythmias, and SCA [1]. We hereby present a case of SCA in a middle aged female caused by anomalous coronary anatomy.

## 2. Case Presentation

**Figure 1:**
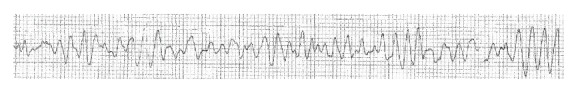
Ventricular fibrillation as the initial rhythm at presentation.

**Figure 2:**
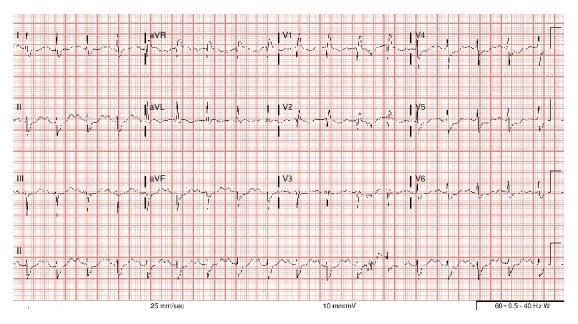
EKG showing sinus rhythm with right bundle branch block, left anterior fascicular block, and nonspecific ST-T wave changes.

**Figure 3:**
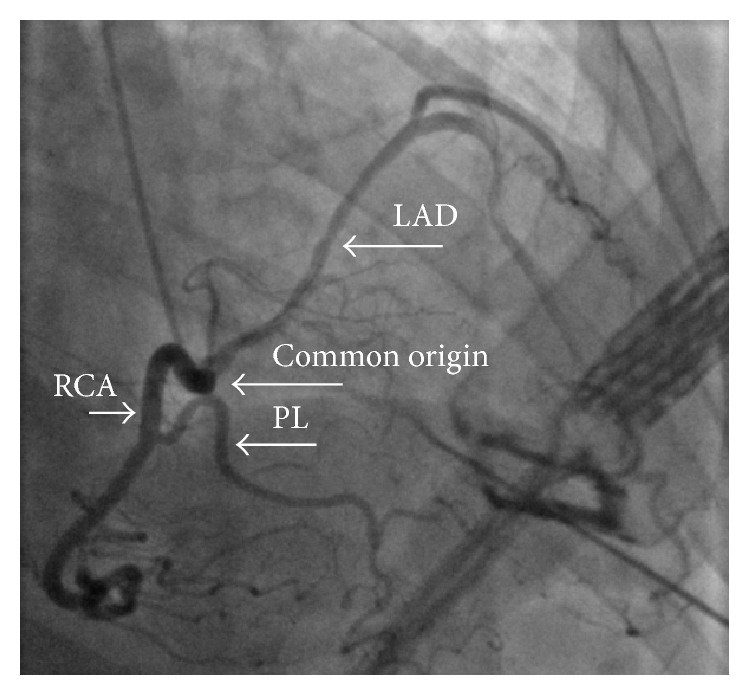
Coronary angiogram showing clear coronaries and anomalous left anterior descending artery originating from the right coronary cusp. RCA: right coronary artery; LAD: left anterior descending artery; PL: posterolateral branch.

**Figure 4:**
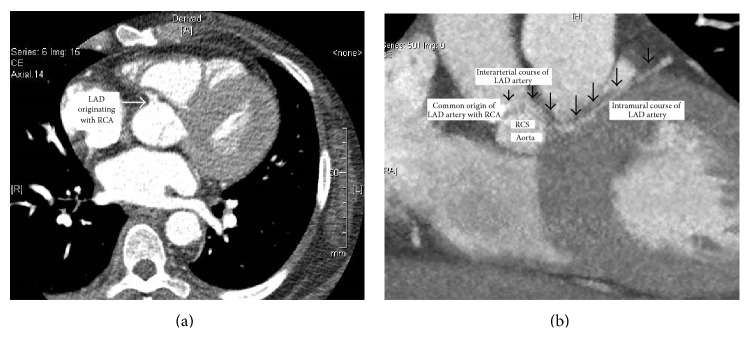
Cardiac coronary CAT scan demonstrating anomalous left coronary artery originating from the right coronary cusp and coursing between aorta and pulmonary artery followed by intramural course. LAD: left anterior descending artery; RCA: right coronary artery.

A 61-year-old African American female with past medical history of unexplained syncope, refractory hypertension, and untreated obstructive sleep apnea was brought to the emergency room (ER) after she experienced a witnessed syncope and became unresponsive at home. When emergency medical service found the patient at home, the presenting rhythm was ventricular fibrillation (Figure 1) and patient was shocked twice with reversal of spontaneous circulation in less than 4 minutes. She was intubated and was taken to the ER. Her presenting blood pressure in the ER was 120/70 mmHg, and heart rate was 114/min. An electrocardiogram (EKG) showed sinus tachycardia, complete right bundle branch block (RBBB) with left anterior fascicular block (LAFB) and nonspecific ST-T wave changes (Figure 2). A bedside echocardiogram showed normal ejection fraction with severe left ventricular hypertrophy and no regional wall motion abnormalities. Initial labs drawn showed mild hypokalemia of 3.2 meq/L (normal value 3.5–5.5 meq/L), glomerular filtration rate of 47 mL/min/1.73 m^2^, normal liver function tests, normal complete blood count, and a serum troponin of <0.04 ng/mL (normal value < 0.04 ng/mL). Coronary angiogram (Figure 3) revealed nonobstructive epicardial coronaries with mildly elevated left ventricular end diastolic pressure (LVEDP) of 21 mmHg. The left coronary artery (LCA) was found to originate from the right coronary sinus. Patient was started on hypothermia protocol. Her troponin started to rise peaking at 4.72 ng/mL. Computerized axial tomography (CAT) scan and magnetic resonance imaging (MRI) scan of head and electroencephalogram were normal. Patient achieved complete neurological recovery in three days. Cardiac coronary CAT scan (Figure 4) was obtained that showed anomalous left anterior descending artery (LAD) originating from the right coronary sinus sharing a common ostium with the right coronary artery (RCA). The artery then had an interarterial course between aorta and pulmonary trunk for 1.7 cm followed by an intramural course for 3.3 cm in the interventricular septum and then exited the myocardium for an epicardial course at the level of mid LAD. The intramural caliber measured 2.2 mm in cross section. The left main coronary artery had a normal origin giving rise to left circumflex and ramus intermedius. Patient underwent one vessel coronary artery bypass grafting with left internal mammary artery to the epicardial LAD at the level immediately after its intramural course. Patient then received implantable cardioverter and defibrillator (ICD) for secondary prevention and was discharged home on a stable condition.

## 3. Discussion

The prevalence of anomalous origin and course of coronary arteries is about 0.7–1.96% [3–5]. This includes a prevalence rate of 0.43% for the RCA branching from the left coronary sinus, the circumflex artery from the RCS or from the RCA, absence of the LMCA, and high takeoff coronary arteries [3]. Incidence of anomalous LAD artery from RCS is 0.03% which is 6 to 10 times less common than the origin of RCA from LCS [1].

Sudden cardiac arrest (SCA) secondary to congenital anomalous coronary artery disease occurs due to insufficient coronary flow by the anomalous LCA to meet elevated left ventricular myocardial metabolic demand, usually during exertion or exercise. In a majority of previously reported cases, SCA was triggered by exertion and most of these patients have a positive exercise stress test [6]. Contributing factors to an increased resistance in the LCA include compression between the great vessels, a slit ostium, myocardial bridging, or unfavorable geometry [7]. High risk defects include those involved with the proximal coronary artery or coursing of the anomalous artery between the aorta and pulmonary trunk [2]. Myocardial bridging refers to intramuscular course of the coronary vessels. Prevalence of myocardial bridging on coronary angiograms has been reported to be less than 5% [8], LAD being the most commonly involved artery.

In the review of 83 angiograms by Dodge Jr. et al., the left main coronary artery was found to be around 4.5 ± 0.5 mm in diameter, the proximal LAD was 3.7 ± 0.4 mm, and the distal LAD measured 1.9 ± 0.4 mm [9]. In our patient, the proximal LAD was buried in the septum with a diameter of 2.2 mm (half of its original diameter) and thus any increase in pressures in the left ventricle during exertion or stress might precipitate ischemia. This could be an explanation for our patient's similar episodes in the past but without sudden cardiac arrest.

Our patient's age was atypical for presentation of sudden cardiac arrest secondary anomalous coronary artery [2]. One possible explanation could be patient's uncontrolled hypertension contributing to progressive left ventricular hypertrophy, which could have caused demand ischemia, precipitating ventricular fibrillation, and SCA. We believe her LV myocardial thickness was not severe enough to precipitate ventricular fibrillation during her previous episodes, thus explaining her atypical presentation at a later age.

Quantitative scar grading with gadolinium MRI helps to assess the extent of infarction and likelihood of myocardial recovery after vascularization [10]. MRI evaluation is comparable and even better in diagnosis of subendocardial infarcts, when compared to PET scan, which is considered as the gold standard for myocardial viability evaluation [11]. In our patient, irrespective of myocardial viability assessment, a revascularization procedure was indicated, considering the episode of SCA and considering the anomalous vessel being LAD.

Documented coronary ischemia in the setting of an anomalous coronary artery coursing between aorta and pulmonary arteries is a class IB indication for surgery according to the American College of Cardiology and American Heart Association (ACC/AHA) guidelines for congenital heart diseases [12]. Exercise stress testing, though commonly employed for diagnosing coronary ischemia, is inadequate in predicting future risk of SCA in patients with anomalous coronaries [13, 14]. Guidelines also recommend surgical correction of anomalous coronary artery coursing between major vessels even in the absence of ischemia [12]. Percutaneous coronary intervention of anomalous intramural coronaries has been associated with poor durability with higher in-stent restenosis rates, coronary artery dissection/rupture, stent fracture, and stent thrombosis [15]. In a study done in pediatric population by Poynter et al., intramural course of the anomalous vessel was found in the majority of the patients who underwent surgery for anomalous coronary artery [16]. The standard surgical technique for treatment of anomalous coronary artery is bypass grafting of the anomalous vessel alone or in combination with native vessel ligation [17], reimplantation of anomalous vessel into appropriate coronary sinus [18, 19], pulmonary artery translocation to increase the space between aorta and pulmonary artery [20], and proximal coronary artery patch enlargement [21]. Most recently, unroofing [22] of the anomalous coronary artery with or without detaching aortic valve commissure has been tried in patients without concomitant atherosclerotic coronary artery disease with favorable results. In our patient, reimplantation was not done because of the intra-arterial and intramural course of the artery, and so an end-to-side bypass of left internal mammary artery to the distal LAD was performed.

Though guidelines recommend ICD implantation in patients with SCA secondary to ventricular arrhythmias [12], there are no guidelines for ICD implantation after surgical correction of anomalous coronary vessel, especially in patients with preserved ejection fraction. In our patient, grafting cannot be done to the proximal LAD because of the long proximal interarterial and intramural course and so she received ICD for secondary prevention of malignant arrhythmias.

Since there is a possible genetic component [23, 24], a transthoracic echocardiogram has traditionally been recommended for first-degree relatives of patients with anomalous coronaries, since these patients will be asymptomatic and their EKG and physical examination will essentially be normal most of the time. Counselling was given to our patient regarding screening family members at the time of her discharge.

We reported a successful surgical repair of anomalous coronary artery causing SCA. Awareness of such presentations is essential among physicians for early recognition and treatment.

## Abbreviations

SCA: Sudden cardiac arrest
LCA: Left coronary artery
LAD: Left anterior descending artery
RCA: Right coronary artery
ICD: Implantable cardioverter and defibrillator.
